# Analgesic and Anti-Inflammatory Effects of the Novel Semicarbazide-Sensitive Amine-Oxidase Inhibitor SzV-1287 in Chronic Arthritis Models of the Mouse

**DOI:** 10.1038/srep39863

**Published:** 2017-01-09

**Authors:** Ádám Horváth, Awt Menghis, Bálint Botz, Éva Borbély, Ágnes Kemény, Valéria Tékus, Janka Zsófia Csepregi, Attila Mócsai, Tamás Juhász, Róza Zákány, Dóra Bogdán, Péter Mátyus, Julie Keeble, Erika Pintér, Zsuzsanna Helyes

**Affiliations:** 1 Department of Pharmacology and Pharmacotherapy, Medical School; János Szentágothai Research Centre & Centre for Neuroscience, University of Pécs, Pécs, Hungary; 2 Institute of Pharmaceutical Science, King’s College London, London, United Kingdom; 3 Department of Medical Biology, Medical School, University of Pécs, Pécs, Hungary; 4 Department of Physiology & MTA-SE 'Lendület' Inflammation Physiology Research Group of the Hungarian Academy of Sciences, Semmelweis University, Budapest, Hungary; 5 Department of Anatomy, Histology and Embryology, Medical School, University of Debrecen, Debrecen, Hungary; 6 Department of Organic Chemistry, Faculty of Pharmacology, Semmelweis University, Budapest, Hungary; 7 PharmInVivo Ltd., Pécs, Hungary; 8 MTA-PTE NAP B Chronic Pain Research Group, Pécs, Hungary

## Abstract

Semicarbazide-sensitive amine oxidase (SSAO) catalyses oxidative deamination of primary amines. Since there is no data about its function in pain and arthritis mechanisms, we investigated the effects of our novel SSAO inhibitor SzV-1287 in chronic mouse models of joint inflammation. Effects of SzV-1287 (20 mg/kg i.p./day) were investigated in the K/BxN serum-transfer and complete Freund’s adjuvant (CFA)-evoked active immunization models compared to the reference SSAO inhibitor LJP-1207. Mechanonociception was assessed by aesthesiometry, oedema by plethysmometry, clinical severity by scoring, joint function by grid test, myeloperoxidase activity by luminescence, vascular leakage by fluorescence *in vivo* imaging, histopathological changes by semiquantitative evaluation, and cytokines by Luminex assay. SzV-1287 significantly inhibited hyperalgesia and oedema in both models. Plasma leakage and keratinocyte chemoattractant production in the tibiotarsal joint, but not myeloperoxidase activity was significantly reduced by SzV-1287 in K/BxN-arthritis. SzV-1287 did not influence vascular and cellular mechanisms in CFA-arthritis, but significantly decreased histopathological alterations. There was no difference in the anti-hyperalgesic and anti-inflammatory actions of SzV-1287 and LJP-1207, but only SzV-1287 decreased CFA-induced tissue damage. Unlike SzV-1287, LJP-1207 induced cartilage destruction, which was confirmed *in vitro*. SzV-1287 exerts potent analgesic and anti-inflammatory actions in chronic arthritis models of distinct mechanisms, without inducing cartilage damage.

Rheumatoid arthritis (RA) is a chronic autoimmune disorder hallmarked by synovitis and progressive joint destruction affecting almost 1% of the population^1^. Although currently used biological therapies are often effective for the inflammation and tissue destruction associated with this disease, joint pain frequently persists and reduces physical activity and life quality^2^. To develop novel pharmacotherapy, intensive research needs to explore the pathophysiological mechanisms of arthritis-related pain. The involvement of neurogenic inflammation in arthritis is well-known, but there is significantly less information about the role of peptidergic afferents and sensory-immune interactions in this condition^3^.

Semicarbazide-sensitive amine oxidase (SSAO), also known as vascular adhesion protein-1 (VAP-1), is a copper containing enzyme which catalyses oxidative deamination of primary amines, resulting in the production of aldehydes, ammonia and hydrogen peroxide^4^. SSAO can be found in a circulating form in the plasma and as a membrane-bound form widely expressed in most tissues and organs, with particularly high levels in endothelial, vascular smooth muscle and adipose cells^5^,^6^. Plasma SSAO activity is significantly elevated in several pathophysiological conditions, such as inflammatory liver diseases, diabetes, congestive heart failure, atherosclerosis, severe obesity and Alzheimer’s disease^7^,^8^,^9^,^10^. After the cloning of VAP-1, which revealed that it is identical to membrane-bound SSAO^11^, substantial evidence has been shown that SSAO plays a prominent, dual role in the transmigration of leukocytes from the bloodstream into inflamed tissues. Studies have shown that small molecule inhibitors of SSAO enzymatic activity impaired leukocyte migration^11^,^12^, suggesting that its locally released, soluble products may promote leukocyte extravasation, acting as direct cross-linkers between immune cells and endothelial cells or promoting the expression of other endothelial surface molecules (e.g., P-selectin, ICAM-1)^13^,^14^,^15^. Several *in vitro* and *in vivo* studies have indicated that SSAO also acts as an important adhesion molecule, since anti-SSAO antibodies disrupt leukocyte-endothelial interactions^11^,^16^,^17^,^18^. Furthermore, SSAO-deficient mice have shown markedly reduced leukocyte recruitment in inflammatory challenges related to models of autoimmune diabetes and peritonitis also supporting the role of SSAO in leukocyte transmigration^19^. Although based on all these data the involvement of SSAO in inflammatory processes is well-established, its potential role in arthritis and pain mechanisms has never been addressed and studied.

Several small molecule SSAO inhibitors have been developed for potential therapeutic purposes, but many of them failed to have an appropriate selectivity and high potency for both experimental and clinical uses. In mammals, SSAO is capable of oxidising biogenic amines (e.g. noradrenaline, dopamine, serotonin etc.), which are also substrates for monoamine oxidase (MAO) A and/or B. Consequently, it is necessary to use inhibitors which are sufficiently selective for SSAO in comparison to MAO, particularly when both enzymes can be found in the same tissue^20^. Furthermore, several compounds possess unfavourable physicochemical properties, such as poor solubility, and they contain certain functional groups (e.g. hydrazine), posing potential toxicity (LJP-1207 and BTT-2052)^21^.

SzV-1287 (3-(4,5-dipheyl-1,3-oxazol-2-yl) propanal oxime) is our newly developed and patented (*PCT WO/2010/015870, PCT P1400205*) SSAO inhibitor^22^,^23^, formed from the oxime analogue of the cyclooxygenase (COX) inhibitor oxaprozin.

SzV-1287 is an innovative metabolism-activated multi-targeting drug, acting by directly inhibiting SSAO activity and simultaneously COX function through its active metabolite^24^. One of the first publications on the effects of this compound reported its anti-inflammatory action in carrageenan-induced acute and adjuvant-induced chronic inflammation models of the rat^25^. We have recently shown that SzV-1287 is also able to antagonise Transient Receptor Potential Vanilloid 1 and Ankyrin 1 (TRPV1 and TRPA1) receptors expressed on nociceptive primary sensory neurons involved in inflammation and pain. These effects were shown to be independent of its inhibitory actions on SSAO^26^.

In the present study, we investigated the effects of our novel compound, SzV-1287, in comparison with a reference SSAO inhibitor on joint inflammation and nociception in chronic arthritis models of distinct mechanisms with complex functional, *in vivo* optical imaging and morphological techniques.

## Results

### SSAO inhibitors reduce K/BxN serum-induced mechanical hyperalgesia and inflammatory signs

**Figure 1:**
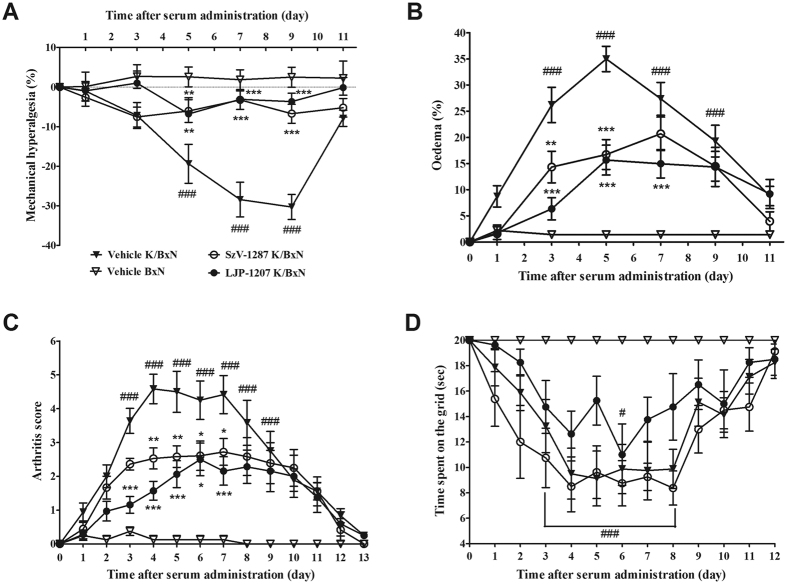
SSAO inhibitors attenuate mechanical hyperalgesia and inflammatory signs in the K/BxN serum-transfer arthritis model. Alterations of the (**A**) mechanonociceptive threshold, (**B**) paw volume, (**C**) semiquantitative clinical score, and (**D**) time spent on the grid in arthritic mice treated with the vehicle, SzV-1287 or LJP-1207 (20 mg/kg/day i.p. every day during the 13-day experimental period) as compared to the BxN serum-injected non-arthritic controls. Data are shown as means ± S.E.M. of n = 8–9 mice/group, *p < 0.05, **p < 0.01, ***p < 0.001 (vs. vehicle-treated arthritic mice), ^#^p < 0.05, ^###^p < 0.001 (vs. vehicle-treated control mice); two-way ANOVA followed by Bonferroni’s multiple comparison test.

In vehicle-treated arthritic mice (n = 9), a 20% decrease in the mechanonociceptive thresholds developed 5 days after the K/BxN serum injection (from 9.17 ± 0.1 g to 7.36 ± 0.42 g), which gradually decreased to 8% by the end of the 21-day study. Repeated daily intraperitoneal (i.p.) treatment with 20 mg/kg SzV-1287 (n = 9) and LJP-1207 (n = 8) similarly and significantly reduced mechanical hyperalgesia in the arthritic groups from the 5^th^ to the 9^th^ day of the experiment (Fig. 1A). Remarkable oedema developed in the ankle and small joints of the hind paw in vehicle-treated arthritic mice (n = 9) reaching a maximal swelling of approximately 35% on day 5 (from 0.18 ± 0.001 cm^3^ to 0.25 ± 0.004 cm^3^). The oedema was significantly attenuated by both SSAO inhibitors, the maximal swelling was approximately 15% in the LJP-1207-treated arthritic group (n = 8) on day 5, and 20% in the SzV-1287-treated group (n = 9) on day 7 (Fig. 1B). Clinical arthritis score reached the maximum value of 4.58 ± 0.44 on day 4 in the vehicle-treated arthritic group (n = 9) and was significantly reduced by both SzV-1287 (n = 9) and LJP-1207 (n = 8) from day 4 and day 3, respectively, till day 7 (SzV-1287 to 2.72 ± 0.39, LJP-1207 to 2.17 ± 0.42) (Fig. 1C). The time spent on the grid as an indicator of grasping ability was reduced, but this parameter was not affected by the treatments (Fig. 1D).

### LJP-1207 significantly reduces neutrophil myeloperoxidase (MPO)-activity in the early phase, whereas both SSAO inhibitors attenuate vascular leakage in the K/BxN serum-transfer arthritis model

**Figure 2:**
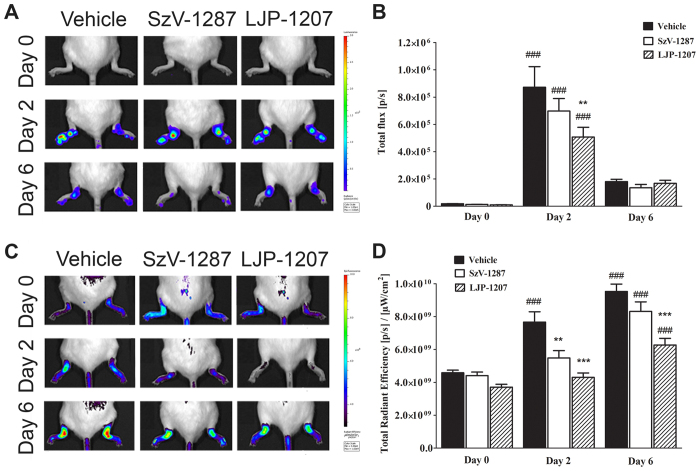
LJP-1207 decreases neutrophil MPO-activity and vascular leakage, SzV-1287 inhibits early plasma protein extravasation in the K/BxN serum-transfer arthritis model. Representative (**A**) bioluminescence images showing neutrophil MPO-activity and (**C**) fluorescence images demonstrating plasma protein extravasation in the tibiotarsal joints of arthritic mice treated with SzV-1287 and LJP-1207 (20 mg/kg/day i.p. every day during the 13-day experimental period) in comparison with the vehicle. Quantitative analysis of (**B**) bioluminescence and (**D**) fluorescence intensity in the ankle joints 2 and 6 days after arthritogenic serum administration. Data are shown as means ± S.E.M. of n = 6 mice/group, **p < 0.01, ***p < 0.001 (vs. vehicle-treated arthritic mice), ^###^p < 0.001 (vs. respective control); one-way ANOVA followed by Bonferroni’s multiple comparison test.

Luminol-derived bioluminescence revealed an increase in neutrophil MPO-activity in the arthritic ankle joints of all groups (n = 6/group), being significantly smaller in the LJP-1207-treated arthritic groups in the early phase as compared to vehicle-treated mice (day 2) (Fig. 2A,B). Plasma leakage, as shown by the fluorescence signal, was significantly smaller in the ankle joints of SSAO inhibitor-treated arthritic groups (n = 6/group) in the early phase. In the late phase (day 6), plasma extravasation enhanced in all groups compared to the early phase, but significant reduction was observed only in LJP-1207-treated mice (Fig. 2C,D).

### SSAO inhibitors decrease CFA-induced mechanical hyperalgesia and oedema

**Figure 3:**
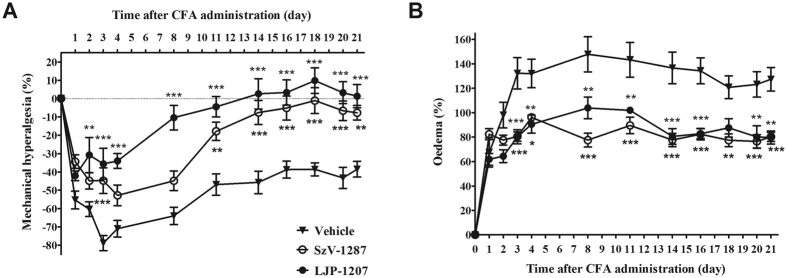
SSAO inhibitors decrease CFA-induced chronic mechanical hyperalgesia and oedema. Changes of the **(A)** mechanonociceptive threshold and **(B)** paw volume in response to CFA injection into the paw and the tail root. The effects of SzV-1287 and LJP-1207 (20 mg/kg/day i.p. every day during the 21-day experimental period) were compared to the vehicle-treated control group. Data are shown as means ± S.E.M. of n = 6–8 mice/group, *p < 0.05, **p < 0.01, ***p < 0.001 (vs. respective limb of vehicle-treated mice); two-way ANOVA followed by Bonferroni’s multiple comparison test.

All groups showed a remarkable decrease of the mechanonociceptive threshold 1 day after CFA-treatment. In vehicle-treated mice (n = 7), a maximal decrease in mechanonociceptive threshold of 79% developed after 3 days (from 8.47 ± 0.26 g to 1.77 ± 0.33 g), gradually decreasing to 40% by the end of the study. This mechanical hyperalgesia was significantly attenuated in both LJP-1207- (n = 6) and SzV-1287-treated mice (n = 8). LJP-1207 showed significant anti-hyperalgesic actions from day 2 to 21, while SzV-1287 on day 3 and in the later phase from day 11 till the end of the experiment (Fig. 3A). The massive, 148%, paw oedema that developed by day 8 in the vehicle-treated group (n = 7) (from 0.18 ± 0.003 cm^3^ to 0.44 ± 0.021 cm^3^) was significantly reduced in both groups treated with SzV-1287 (n = 8) or LJP-1207 (n = 6) throughout the whole experimental period (Fig. 3B).

### SSAO inhibitors do not influence the neutrophil MPO-activity and vascular leakage in the tibiotarsal joints on the CFA-injected side

**Figure 4:**
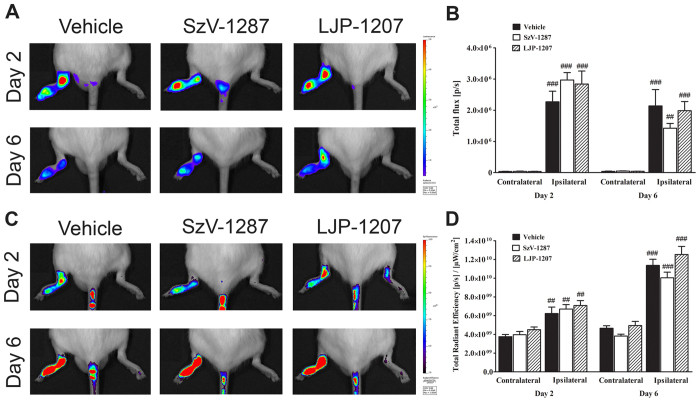
SSAO inhibitors do not alter CFA-evoked neutrophil MPO-activity and plasma extravasation. Representative **(A)** bioluminescence images showing neutrophil MPO-activity and **(C)** fluorescence images demonstrating plasma protein extravasation in the tibiotarsal joints of mice treated with SzV-1287 and LJP-1207 (20 mg/kg/day i.p. every day during the 21-day experimental period) in comparison with the vehicle. Quantitative analysis of **(B)** bioluminescence and **(D)** fluorescence intensity in the ankle joints 2 and 6 days after CFA administration. Data are shown as means ± S.E.M. of n = 6 mice/group, ^##^p < 0.01, ^###^p < 0.001 (vs. respective contralateral ankle joint); one-way ANOVA followed by Bonferroni’s multiple comparison test.

All groups (n = 6/group) showed intensive luminol-derived bioluminescence signal in the tibiotarsal joints on the CFA-injected side, but it was not significantly different between any of the groups either on day 2 or day 6 (Fig. 4A,B). Plasma leakage was similarly high in the arthritic ankle joints of all groups (n = 6/group) in the early phase, which increased further by day 6. However, significant difference was not observed between the groups (Fig. 4C,D).

### SSAO inhibitors reduce the production of keratinocyte chemoattractant (KC), but not other inflammatory cytokines in the arthritic tibiotarsal joints

**Figure 5:**
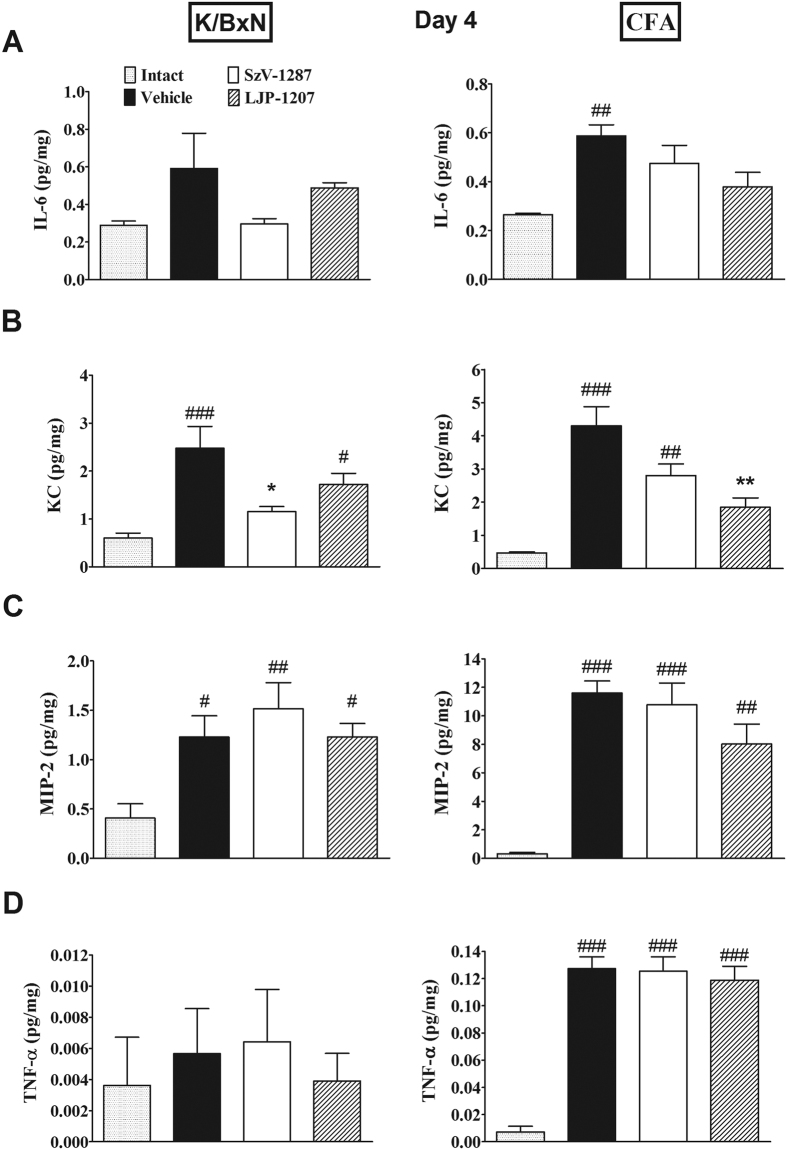
SSAO inhibitors decrease arthritis-induced KC, but not other inflammatory cytokine production in the tibiotarsal joints. Concentrations of (**A**) IL-6, (**B**) KC, (**C**) MIP-2 and (**D**) TNF-α in the homogenates of the tibiotarsal joints of vehicle-, SzV-1287- and LJP-1207-treated (20 mg/kg/day i.p. every day during the 4-day experimental period) arthritic mice in comparison with non-arthritic (BxN) or intact mice. Data are means ± S.E.M. of n = 4–6 mice/group, *p < 0.05, **p < 0.01 (vs. respective vehicle-treated group), ^#^p < 0.05, ^##^p < 0.01, ^###^p < 0.001 (vs. non-arthritic or intact mice); one-way ANOVA followed by Bonferroni’s multiple comparison test.

In the K/BxN-serum-transfer arthritis model the concentrations of chemokine (C-X-C motif) ligand 1 (CXCL1) also called KC and chemokine (C-X-C motif) ligand 2 (CXCL2) also called macrophage inflammatory protein 2 (MIP-2) significantly increased in the ankle joint homogenates of vehicle-treated arthritic mice (n = 5) on day 4 compared to non-arthritic animals (n = 6). Meanwhile, interleukin-6 (IL-6) and tumor necrosis factor-alpha (TNF-α) levels did not elevate significantly in the arthritic joints of vehicle-treated mice at this time point. SzV-1287 (n = 5) significantly reduced exclusively the inflammation-evoked tissue KC elevation, but LJP-1207 treatment (n = 5) only showed an inhibitory tendency (Fig. 5A–D).

In comparison, the production of all the measured inflammatory cytokines were remarkably greater in the CFA model. The concentrations of IL-6, KC, MIP-2 and TNF-α in the tibiotarsal joint homogenates of vehicle-treated mice (n = 5) significantly increased on the CFA-injected side on day 4 compared to intact animals (n = 4). Despite what was found in K/BxN serum-transfer arthritis, KC increase was significantly reduced by LJP-1207 (n = 5) in this model, and SzV-1287 administration (n = 5) only resulted in a decreasing tendency. The inflammation-evoked increase of the other cytokines were not influenced by either compounds (Fig. 5A–D).

Interleukin-1beta (IL-1β) in the joint homogenates and any of the 5 investigated cytokines in the plasma did not change in either models or in any of the groups at this time point (data not shown).

### SzV-1287 reduces histopathological severity of arthritis

**Figure 6:**
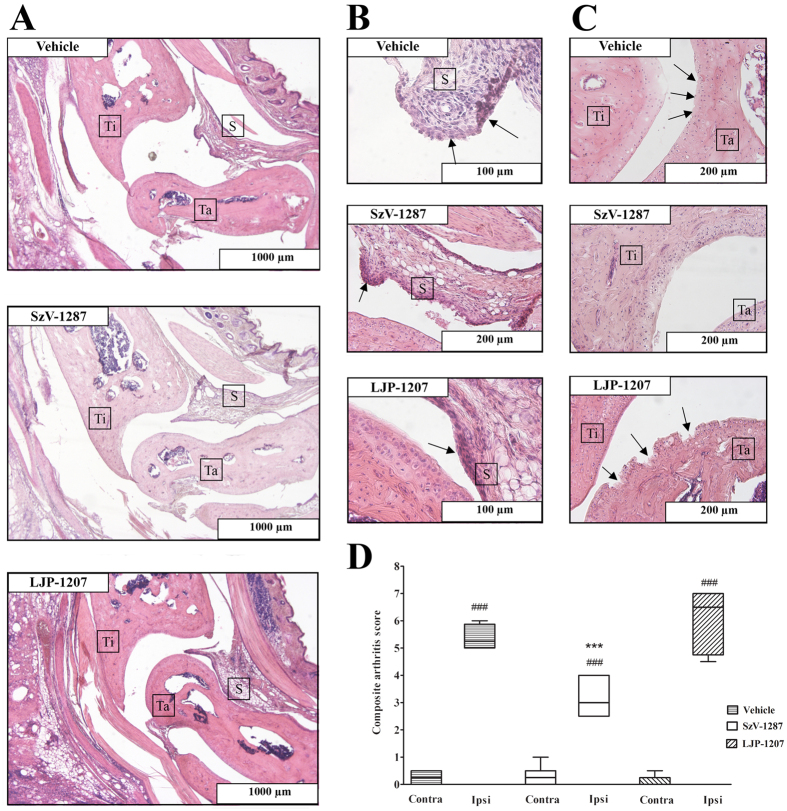
SzV-1287 decreases histopathological alterations in the tibiotarsal joint in response to CFA injection. Representative slides stained with hematoxylin-eosin of **(A–C)** the tibiotarsal joint of vehicle-, SzV-1287- and LJP-1207-treated (20 mg/kg/day i.p. every day during the 21-day experimental period) arthritic mice. (A: 40x, B (Vehicle, LJP-1207): 400x, B (SzV-1287): 200x, C (Vehicle, LJP-1207): 200x, C (SzV-1287): 100x magnification, Ti = Tibia bone, Ta = Tarsal bone, S = Synovium). **(D)** Semiquantitative histopathological scoring on the basis of inflammatory cell infiltration, synovial hyperplasia, cartilage destruction and bone erosion. Box plots shows medians of composite scores of n = 4–8 mice/group, ***p < 0.001 (vs. ipsilateral ankle joint of vehicle-treated mice), ^###^p < 0.001 (vs. respective contralateral ankle joint); one-way ANOVA followed by Bonferroni’s multiple comparison test.

Histopathological evaluation of the left tibiotarsal joints (CFA-injected side) of the vehicle treated animals (n = 4) revealed synovial lining hyperplasia, infiltration of mononuclear cells into the synovium and cartilage destruction, which are all characteristic histopathological alterations in arthritis. Mice treated with SzV-1287 (n = 8) showed markedly reduced inflammatory cell infiltration into the synovium and minimal damage of the cartilage, only synovial cell lining hyperplasia was similar to controls. Animals treated with LJP-1207 (n = 5) showed slightly reduced infiltration of inflammatory cells into the synovium, compared to vehicle-treated mice, but showed substantial cartilage destruction and synovial cell lining hyperplasia (Fig. 6A–C). Semiquantitative scoring and composite arthritis scores revealed that arthritis severity was only significantly decreased in SzV-1287-treated animals, compared to the vehicle-treated group (Fig. 6D).

### LJP-1207, but not SzV-1287 administration decreases the metachromatic matrix production in micromass cell cultures

**Figure 7:**
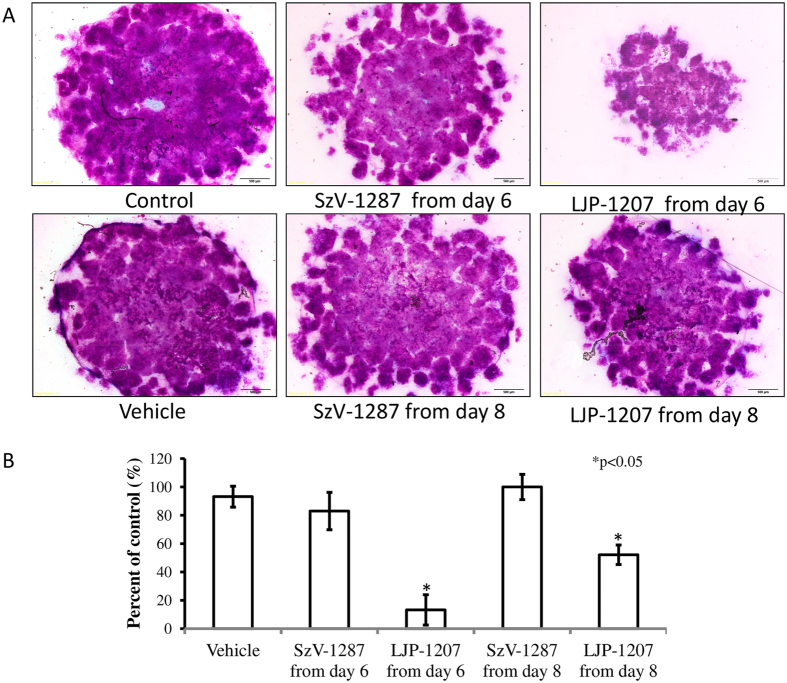
Effects of SzV-1287 and LJP-1207 on matrix production of chondrocyte cultures. SzV-1287 at 10 μM and LJP-1207 at 10 μM were added to the culture medium and were present continuously started from day 6 or day 8. (**A**) Representative picture of the metachromatic cartilage areas in 10-day-old micromass cultures were visualised with DMMB dissolved in 3% acetic acid. Metachromatic (purple) areas represent cartilaginous nodules formed by many chondrocytes embedded into a cartilage matrix rich in polyanionic glycosaminoglycan (GAG) chains. Original magnification was 4x. Scale bar, 500 μm. (**B**) Optical density (OD625) was determined in samples containing TB extracted from micromass cultures with 8% HCl dissolved in absolute ethanol. Asterisks indicate significant (*p < 0.05) alterations of the amount of extracted TB (reflecting on the amount of GAG-rich matrix) as compared to the respective control. Representative data of 3 independent experiments exhibiting the same patterns of changes are shown.

In line with our *in vivo* results, SzV-1287 exerted only a moderate effect on cartilage matrix production *in vitro*. No significant differences were detected on day 10 between the cultures treated with SzV-1287 continuously starting from day 6 or 8 as compared to vehicle. In contrast, the application of LJP-1207 significantly decreased the amount of metachromatic cartilage matrix produced in chicken micromass cultures by day 10, following initiation of treatment at days 6 and 8, as revealed by both dimethyl methylene blue (DMMB) and toluidine blue (TB) staining (Fig. 7A,B).

## Discussion

We provide the first data on potent analgesic and anti-inflammatory effects of our novel SSAO inhibitor, SzV-1287, in chronic mouse arthritis models of different mechanisms (Table 1). The K/BxN serum-transfer arthritis model is a widely used passive transfer mouse model of RA, while the adjuvant arthritis model is mediated by active immunization. The K/BxN serum-transfer arthritis model shares many immunological, histological and clinical features with RA including activation of neutrophils, macrophages, mast cells and complement system, inflammatory cytokine production (e.g. IL-1, TNF-α), erosive synovitis^27^. The dynamics of the pathophysiological alterations are also similar to the human disease. The early phase is characterised by a transient inflammation with swelling of the small joints of all paws and mechanical hyperalgesia which is resolved in after 10–14 days, if the serum injection is not repeated. However, even following the resolution of the inflammatory phase, tactile hypersensitivity persists^28^. This is similar to the clinical conditions, where although severe swelling and joint destruction are successfully treated with the novel biologics, pain still remains an important therapy-resistant problem. In comparison, CFA induces a destructive arthritis, often used to examine the effects of immunomodulatory and anti-inflammatory drugs^29^,^30^,^31^. This model exhibits a biphasic time course, comprising of an acute phase (3–5 days) followed by a chronic inflammation resulting in several signs of joint deformation (synovial hyperplasia, angiogenesis, pannus formation, capsular fibrosis, cartilage destruction, bone erosion and bone matrix resorption) and inflammatory mechanisms (T-cell dominant immune response, increased levels of TNF-α, IL-6, IL-7, IFNγ and IL-1β) associated with RA^32^,^33^.

In the K/BxN serum-transfer arthritis model, our novel compound, SzV-1287, significantly reduced mechanical hyperalgesia, swelling and plasma extravasation in the early phase at a similar extent to the reference SSAO inhibitor, LJP-1207. Both compounds also showed similar significant anti-hyperalgesic and anti-oedema actions in the CFA model, from day 3, but there were no significant effects on neutrophil MPO-activity and plasma extravasation. Among the 5 characteristic inflammatory cytokines (IL-β, IL-6, KC, MIP-2, TNF-α) in the arthritic tibiotarsal joints on day 4 when previous data showed their increase^34^, only the inflammation-induced increased production of KC was significantly reduced by the SSAO inhibitors. However, the inhibitory effect of SzV-1287 reached the level of statistical significance in the K/BxN serum-transfer arthritis model and that of LJP-1207 in the CFA model, they exerted a similar action on this inflammatory parameter. KC is synthesised by macrophages, endothelial cells and synoviocytes, it is an important chemoattractant and increases neutrophil recruitment^35^,^36^,^37^. These data suggest that the anti-inflammatory and anti-hyperalgesic actions of SSAO inhibitors are not predominantly related to cytokine production inhibition, although it might have a partial role through decreasing the synthesis of KC.

Although we are the first to report the analgesic actions of SSAO inhibition, the described anti-inflammatory effects are in agreement with previous data showing anti-oedema effects for SzV-1287 in carrageenan- and CFA-induced paw inflammation models in the rat^25^. However, the most remarkable difference between the effects of the two compounds was the significant reduction in the inflammatory histopathological alterations (mononuclear cell infiltration and cartilage damage) in SzV-1287-treated mice, while chronic LJP-1207 treatment actually worsened the cartilage destruction at the 20 mg/kg i.p. dose, resulting in similarly worsened therapeutic outcomes. This *in vivo* observations were supported by the decreased metachromatic cartilage matrix production detected after LJP-1207, but not SzV-1287 treatment, in chondrocyte cultures. The cartilage damaging action of LJP-1207 in the chronically inflamed joint is not likely to be due to its SSAO inhibitory effect, since a very recently published paper reports that SSAO inhibition delayed the chondrocyte differentiation without any alteration of cell viability *in vitro*^38^; this might be related to some other mechanisms due to its different structure. The unique features of SzV-1287 compared to other SSAO inhibitors are its COX inhibitory action, without ulcerogenic effects^25^, and potent dual antagonistic property at the TRPV1/TRPA1 ion channels localised on peptidergic sensory nerves^39^, inflammatory cells^40^, and chondrocytes^41^,^42^. The importance of TRPV1/TRPA1-dependent neurogenic inflammatory mechanisms in RA has been extensively investigated and received a great attention^43^,^44^,^45^. Both receptors are a ligand-gated cation channel activated by various inflammatory mediators including protons, lipids, reactive oxygen species and lipoxygenase products, which result in the release of a broad range of neuropeptides^40^. The TRPV1/TRPA1 antagonistic actions of SzV-1287 may explain its beneficial effect against cartilage destruction in our models, which is supported by recent data showing a potent protective action of TRPA1 activation on human and mouse chondrocytes, against monosodium iodoacetate (MIA)-evoked inflammatory damage^42^.

Although LJP-1207 showed greater potency in the inhibition of human SSAO activity in an *in vitro* enzyme assay compared to SzV-1287 (IC_50_: 1.7 × 10^−8^ M and IC_50_: 3.5 × 10^−6^ M, respectively)^25^, there was no significant difference between their actions in our chronic *in vivo* experiments. The described anti-inflammatory effects are novel in relation to arthritis, but they are explained by the already known vascular and cellular mechanisms of SSAO inhibition and supported by previous literature^11^,^16^,^17^,^18^. However, these are the first data in relation to pain, which may be related to the inhibition of both peripheral and central sensitization processes of the nociceptive pathway. The latter effect is supported by some publications showing SSAO activity in the mouse brain both in the endothelial cells of microvessels and neurons^46^,^47^. Despite the fact that a range of anti-inflammatory effects LJP-1207 have been shown in several inflammation models including colitis, multiple sclerosis, LPS-induced endotoxemia and stroke^48^,^49^, it is not a suitable compound for drug development, because as a hydrazine derivative it is potentially toxic upon repeated exposure^21^,^50^.

In conclusion, these are the first data describing that SSAO inhibition exerts analgesic effects. We have shown that our novel SSAO inhibitor, SzV-1287, with a unique complex mechanism of action inhibits chronic arthritis with special emphasis on related pain and cartilage destruction. Therefore, it opens promising perspectives for the development of a novel analgesic and anti-inflammatory drug.

## Methods

### Experimental animals

Experiments were performed on 8–12-week-old male CD1 mice (30–40 g) provided with standard chow and water ad libitum under a 12-hour light/dark cycle. They were bred and kept in the Laboratory Animal House of the Department of Pharmacology and Pharmacotherapy, University of Pécs in 160x137 × 330 mm sized cages with 5–10 mice/cage density at 24–25 °C. During the experiments, they were kept in groups with their littermates. The total number of animals used in the experiments were 95 and they were allocated to experimental groups with randomised section. The animal numbers per group were estimated on the basis of previous experiments carried out with similar methodology. In behavioural testing, due to the expected higher variability at least 6 mice/group were necessary to use to detect statistically significant differences. We tried to minimise the numbers of experimental animals using same mice for functional measurements, for the *in vivo* imaging and also for the histopathological evaluation.

### Ethics statement

All procedures were performed according to the 1998/XXVIII Act of the Hungarian Parliament on Animal Protection and Consideration Decree of Scientific Procedures of Animal Experiments (243/1988), complied with the recommendations of the International Association for the Study of Pain. Ethical approval was given by the Ethics Committee on Animal Research of University of Pécs according to the Ethical Codex of Animal Experiments (licence No.: BA 02/2000–2/2012).

### Drugs

SzV-1287 and LJP-1207 were both synthesised at the Department of Organic Chemistry, Semmelweis University, Budapest, Hungary as previously described^22^,^48^. LJP-1207 was dissolved in distilled water, while SzV-1287 in a vehicle containing 2% Tween, 2% ethanol and 96% distilled water. Vehicle-treated mice served as controls. Solutions were prepared immediately before use and injected i.p. at a volume of 0.1 ml/10 g (2 mg/ml solution; 20 mg/kg dose) every day throughout the 13- and 21-day experimental periods. This dose was chosen based on the results obtained earlier in acute paw inflammation^25^ and chronic pain models of neuropathic components (*PCT P1400205*)^23^.

### K/BxN serum-transfer model of rheumatoid arthritis

Arthritogenic K/BxN and control BxN sera were obtained from KRN T cell receptor (TCR) transgene-positive (K/BxN) and negative (BxN) mice^51^. TCR in the K/BxN mice recognises a ubiquitously expressed self-protein, glucose-6-phosphate isomerase, inducing an autoimmune response and severe inflammatory arthritis^52^,^53^. Arthritis was induced by 300 μl i.p. K/BxN (n = 26) or BxN serum (n = 8) on day 0^54^. Daily i.p. drug (20 mg/kg SzV-1287, n = 9; 20 mg/kg LJP-1207, n = 8) and vehicle treatment (n = 17) started 20 minutes prior to serum-injection and continued for 13 days. I.p. injections were always performed 20 min before the mechanonociceptive testing or plethysmometry, or on days when both measurements were done, the mechanonociceptive thresholds were measured first followed directly by the paw volume determination. The treatments and measurements were done in 5-minutes intervals. In the *in vivo* imaging studies they were administered simultaneously to 2 mice at a time window of 20 minutes, since the whole measurement takes about 20 minutes and 2 mice can be measured in the chamber of the equipment at the same time. Ankle oedema, hyperalgesia, joint function, clinical inflammation severity, plasma leakage and myeloperoxidase activity were assessed repeatedly *in vivo* during the 2-week experimental period, as described below.

### CFA-induced chronic inflammation model

Complete Freund’s adjuvant (CFA) consisting of heat-killed Mycobacterium tuberculosis suspended in paraffin oil (1 mg/ml; Sigma-Aldrich, St. Louis, MO, USA) induces chronic inflammation. Mice were injected with 20–20 μl of CFA intraplantarly into the left paw and subcutaneously (s.c.) into the tail root (n = 21). Additional s.c. injection into the tail was administered after 24 hours to potentiate the systemic effects mimicking the human condition^55^. Daily i.p. drug (20 mg/kg SzV-1287, n = 8; 20 mg/kg LJP-1207, n = 6) and vehicle treatment (n = 7) started 20 min before CFA-injections on day 0 and day 1, then continued for 21 days. From day 2 SSAO-inhibitors were injected the same way as described above for the K/BxN model. Ankle oedema, hyperalgesia, plasma leakage and MPO-activity were assessed *in vivo* during the 3-week experimental period, and histopathological evaluation was performed from the tibiotarsal joints excised at the end of the study, as described below.

### Measurement of touch sensitivity of the paw

Touch sensitivity of the hind paw was evaluated using dynamic plantar aesthesiometry (DPA, Ugo Basile 37400, Comerio, Italy) prior to and 1, 2, 3, 4, 8, 11, 14, 16, 18, 20 and 21 days after CFA injection, 1, 3, 5, 7, 9 and 11 days after K/BxN injection, with a maximum force of 10 g with 4 second latency. Mechanical hyperalgesia was expressed as a percentage decrease compared to the baseline (day 0) mechanonociceptive threshold values^55^.

### Measuring paw volume

Paw swelling was measured using plethysmometry (Ugile Basile Plethysmometer 7140, Comerio, Italy) prior to and 1, 2, 3, 4, 8, 11, 14, 16, 18, 20 and 21 days after CFA injection, 1, 3, 5, 7, 9 and 11 days after K/BxN injection, it was expressed as a percentage change compared to the baseline (day 0) paw volume^55^.

### Assessment of joint function (grid test)

The grasping ability correlating with joint function was determined using the grid test in the K/BxN serum-transfer arthritis model, in which the small joints of all the four limbs are affected. Mice were placed on a horizontal wire grid, then it was turned over and the latency to fall was determined. The grid was maintained in horizontal position for a maximum of 20 seconds. The grid test was performed daily for 12 days following serum injection^54^.

### Evaluation of arthritis severity

The classical signs of the inflammation, oedema and hyperaemia on both hind limbs, were evaluated with semiquantitative clinical scoring (0–1.5: healthy, 1.5–2.5: minimal signs referred to disease, 2.5–4: mild inflammation, 4–7: moderate inflammation, 7–10: severe inflammation) in the K/BxN serum-transfer arthritis model^54^. This semiquantitative analysis of inflammation is an appropriate supplementation of the paw volume measurement, since it does not only give information about swelling, but also about hyperaemia, so we can get a more complex view of the disease severity. Furthermore, this rapid evaluation method is useful for selecting the appropriate time points for more complex functional tests. Scores were assessed before serum injection and every day during the 13 day-experimental period.

### *In vivo* optical imaging

Luminol (5-amino-2,3-dihydro-1,4-phthalazine-dione) sodium salt (Gold Biotechnology, Olivette, MO, USA) was used to detect reactive oxygen species (ROS) and neutrophil MPO-activity, IR-676 vascular fluorescent dye (Spectrum-Info Ltd., Kyiv, Ukraine) to evaluate capillary leakage^56^,^57^. Animals were anaesthetised using ketamine (100 mg/kg; Calypsol, Gedeon Richter Plc., Budapest, Hungary) and xylazine (10 mg/kg; Sedaxylan, Eurovet Animal Health B.V., Bladel, Netherlands), hair was removed from both hind legs to prevent scattering/absorbing of light signal. Luminol (150 mg/kg) in sterile phosphate-buffered saline (PBS, 20 mg/ml) was injected i.p., IR-676 (0.35 mg/kg) in Kolliphor HS 15 (polyethylene-glycol-15-hydroxystearate; Sigma-Aldrich, St. Louis, MO, USA) intravenously (i.v.) 2 and 6 days following CFA (n = 6/group) or serum administration (n = 6/group). Bioluminescence was measured 10 min, fluorescence 20 min post-injection using the IVIS Lumina II (PerkinElmer, Waltham, USA; 60 s aquisition, F/stop = 1, Binning = 8 in bioluminescence; auto acquisition time, F/stop = 1, Binning = 2, excitation: 745 nm, emission filter: >800 nm in fluorescence). Data were analysed using the Living Image® software (Perkin-Elmer, Waltham, USA). Identical Region of Interests (ROIs) were applied around both ankles, calibrated units of the luminescence (total radiance (total photon flux/s)) and the fluorescence (total radiant efficiency ([photons/s/cm^2^/sr]/[μW/cm^2^])) originating from the ROIs were analysed^58^.

### Measurement of inflammatory cytokine concentrations in the tibiotarsal joint homogenates and plasma

Mice were deeply anaesthetised with sodium pentobarbital (1%; 0.07 ml/10 g) on the 4^th^ day after CFA- or K/BxN serum administration. Blood samples were taken by cardiac puncture into ice-cold tubes containing 40 μl of 50 mg/ml pH 7.5 EDTA (Reanal, Budapest, Hungary) and 20 μl of 10.000 KIU/ml aprotinin (Gordox, Gedeon Richter Plc., Budapest, Hungary), and centrifuged for 5 min at 1000 rpm and for 10 min at 10.000 rpm. The excised tibiotarsal joints were weighted, immediately placed in ice-cold PBS containing 10 mg/ml phenylmethanesulfonyl fluoride (PMSF; Sigma-Aldrich, St. Louis, MO, USA) protease inhibitor, homogenized on ice with Miccra D-9 Digitronic device (Art-moderne Labortechnik, Müllheim, Germany) and centrifuged for 10 min (4 °C, 12.500 rpm). The plasma and the supernatants of the homogenates were stored at −80 °C until the measurements^59^.

Luminex Multiplex Immunoassay was performed to determine the concentrations of 5 characteristic inflammatory cytokines using customized Milliplex Mouse Cytokin/Chemokine Magnetic Bead Panel (Merck Millipore, Billerica, MA, USA): IL-1β, IL-6, KC, MIP-2, TNF-α. Following previous optimizations, all samples were tested undiluted in a blind-fashion in duplicate. The experiment was performed according to the manufacturer’s instructions. Briefly, 25 μl volume of each sample, control and standard were added to a 96-well plate containing 25 μl of capture antibody coated, fluorescent-coded beads. Biotinylated detection antibodies and streptavidin-PE were added to the plate after the appropriate incubation periods. After the last washing step, 150 μl volume of sheath fluid was added to the wells, the plate was incubated and read on the Luminex100 instrument. Five-PL regression curve were used to plot the standard curves for all analyte by the xPonent 3.1 software analysing the bead median fluorescence intensity.

### Histology

Animals were euthanised using sodium pentobarbital (1%; 0.07 ml/10 g) on day 21 in the CFA model, where arthritic morphological changes develop. The tibiotarsal joints were fixed in 4% buffered formaldehyde, decalcified and paraffin-embedded, sliced into sections (5 μM) and stained with haematoxylin and eosin. Arthritic changes were scored by a blinded observer using a scale of 0 to 3 according to 1) mononuclear cells infiltration into areolar tissue, 2) synovial hyperplasia, 3) cartilage destruction, 4) bone erosion^55^. Sections obtained from 4 mice were evaluated in the vehicle-treated group, 8 in the SzV-1287-treated group, 5 in the LJP-1207-treated group.

### Cell culturing

Chondrifying primary micromass cell cultures were made from chicken embryos (Ross hybrid) of Hamburger-Hamilton stages 22–24^60^. Distal parts of the limb buds were removed and droplets from cell suspensions (15 × 10^6^ cell/ml) were inoculated on round coverglasses (Menzel-Gläser, Menzel GmbH, Braunschweig, Germany) placed into the wells of 24-well culture plates (Eppendorf, Hamburg, Germany) on day 0. Colonies were fed with Ham’s F12 medium (Sigma-Aldrich, St. Louis, MO, USA), supplemented with 10% foetal calf serum (Gibco, Gaithersburg, MD, USA) and were kept at 37 °C in the presence of 5% CO_2_ and 80% humidity in a CO_2_ incubator. Cultures were maintained for 10 days, the micromass cultures were mostly differentiated by day 6, matured hyaline cartilage is suitable to investigate the *in vitro* effects of compounds on cartilage. Drugs were were applied to the culture medium continuously from day 6 or from day 8 in 10 μM final concentrations, their vehicles served as controls.

### Metachromatic staining

Cell cultures were fixed in 4:1 mixture of absolute ethanol and 40% formaldehyde on day 10 and stained with 0.1% DMMB (Sigma-Aldrich, St. Louis, MO, USA) dissolved in 3% acetic acid. After washing in 3% acetic acid, cultures were mounted in gum Arabic. Photomicrographs were taken using an Olympus DP72 camera on a Nikon Eclipse E800 microscope (Nikon Corporation, Tokyo, Japan). For semiquantitative detection of the metachromatic matrix we dissolved back TB (pH 2; Reanal, Budapest, Hungary) from cell cultures on day 6, which provides a good approximation of the amount of formed cartilage^61^. DMMB and TB metachromatic staining procedures were carried out on separate colonies from the same experiments. The absorbance of the metachromatic areas was measured in 3 cultures of each group in 3 independent experiments.

### Statistical analysis

Data were analysed using GraphPad Prism 5 and tested for normal distribution using Shapiro-Wilk normality test. Results were expressed as means ± standard errors of means (S.E.M.) and box plots in case of the histopathological evaluation representing the median composite scores. Statistical analysis was performed by repeated measures two-way analysis of variance (ANOVA) followed by Bonferroni’s multiple comparison test in cases of hyperalgesia, oedema, clinical scoring and grid test results, while one-way ANOVA followed by Bonferroni’s post-hoc test for multiple comparisons in cases of the optical imaging, cytokine concentrations and histopathological scoring data. The TB data were analysed by unpaired Student’s t-test. The significance level was set at p < 0.05.

## Additional Information

**How to cite this article**: Horváth, Á. *et al*. Analgesic and anti-inflammatory effects of the novel semicarbazide-sensitive amine-oxidase inhibitor SzV-1287 in chronic arthritis models of the mouse. *Sci. Rep.* **7**, 39863; doi: 10.1038/srep39863 (2017).

**Publisher's note:** Springer Nature remains neutral with regard to jurisdictional claims in published maps and institutional affiliations.

## Figures and Tables

**Table 1:** Summary of the effects of SSAO inhibitors in mouse arthritis models of distinct mechanisms.

	CFA model		K/BxN model	
	SzV-1287	LJP-1207	SzV-1287	LJP-1207
Mechanical hyperalgesia	↓	↓	↓	↓
Joint swelling	↓	↓	↓	↓
Semiquantitative score	n.a.	n.a.	↓	↓
Joint function	n.a.	n.a.	Ø	Ø
Neutrophil MPO-activity	Ø	Ø	Ø	↓
Plasma extravasation	Ø	Ø	↓	↓
Histopathological changes	↓	Ø	n.a.	n.a.

Both SzV-1287 and LJP-1207 significantly reduced CFA- and K/BxN serum-evoked mechanical hyperalgesia and joint swelling, as well as K/BxN serum-induced macroscopic clinical sings and plasma extravasation. K/BxN serum-evoked neutrophil MPO-activity was attenuated only by LJP-1207, CFA-induced histopathological destruction by SzV-1287. K/BxN serum-induced joint function impairment, CFA-evoked neutrophil MPO-activity and plasma extravasation were not affected by either treatment (↓: significantly reduced; Ø: not affected; n.a.: not analysed).
